# An Automated Field Phenotyping Pipeline for Application in Grapevine Research

**DOI:** 10.3390/s150304823

**Published:** 2015-02-26

**Authors:** Anna Kicherer, Katja Herzog, Michael Pflanz, Markus Wieland, Philipp Rüger, Steffen Kecke, Heiner Kuhlmann, Reinhard Töpfer

**Affiliations:** 1 Julius Kühn-Institut, Federal Research Centre of Cultivated Plants, Institute for Grapevine Breeding Geilweilerhof, 76833 Siebeldingen, Germany; E-Mails: katja.herzog@jki.bund.de (K.H.); reinhard.toepfer@jki.bund.de (R.T.); 2 Julius Kühn-Institut, Federal Research Centre for Cultivated Plants, Institute for Plant Protection in Field Crops and Grassland, Messeweg 11-12, 38104 Braunschweig, Germany; E-Mail: mpflanz@atb-potsdam.de; 3 Leibniz Institute for Agricultural Engineering Potsdam-Bornim, Department Horticultural Engineering, Max-Eyth-Allee 100, 14469 Potsdam, Germany; 4 University of Bonn, Department of Geodesy, Institute for Geodesy and Geoinformation (IGG), Nussallee 17, 53115 Bonn, Germany; E-Mails: m.wieland@uni-bonn.de (M.W.); heiner.kuhlmann@uni-bonn.de (H.K.); 5 Geisenheim University, Department of Viticultural Engineering, Brentanostraße 9, 65366 Geisenheim, Germany; E-Mail: Philipp.Rueger@hs-gm.de; 6 Julius Kühn-Institut, Federal Research Centre for Cultivated Plants, Department of Data Processing, Erwin-Baur-Str. 27, 06484 Quedlinburg, Germany; E-Mail: steffen.kecke@jki.bund.de

**Keywords:** robot, geoinformation, high-throughput analysis, image acquisition, plant phenotyping, grapevine breeding, *Vitis vinifera*

## Abstract

Due to its perennial nature and size, the acquisition of phenotypic data in grapevine research is almost exclusively restricted to the field and done by visual estimation. This kind of evaluation procedure is limited by time, cost and the subjectivity of records. As a consequence, objectivity, automation and more precision of phenotypic data evaluation are needed to increase the number of samples, manage grapevine repositories, enable genetic research of new phenotypic traits and, therefore, increase the efficiency in plant research. In the present study, an automated field phenotyping pipeline was setup and applied in a plot of genetic resources. The application of the *PHENObot* allows image acquisition from at least 250 individual grapevines per hour directly in the field without user interaction. Data management is handled by a database (*IMAGEdata*). The automatic image analysis tool *BIVcolor* (Berries in Vineyards-color) permitted the collection of precise phenotypic data of two important fruit traits, berry size and color, within a large set of plants. The application of the *PHENObot* represents an automated tool for high-throughput sampling of image data in the field. The automated analysis of these images facilitates the generation of objective and precise phenotypic data on a larger scale.

## 1. Introduction

With the fast development of genotyping methods to support grapevine breeding based on SSR (Simple Sequence Repeats) [1,2] or SNP (Single Nucleotide Polymorphism) analyses, including next generation DNA sequencing [3], genotyping efficiency has been greatly improved and costs have been reduced contemporaneously. However, plant phenotyping methods have only slowly improved during the last few decades, becoming now a major bottleneck. Therefore, the lack of sufficient phenotypic data and phenotyping methods constrains the possibility to reveal the genetics of quantitative traits, such as yield, growth and adaption to abiotic or biotic stresses. The development and implementation of high-throughput phenotyping platforms is therefore a key tool to improve the efficiency of grapevine (*Vitis vinifera* L. subsp. *vinifera*) or, more generally, plant breeding. In recent years, much effort has been made to build up such platforms, which allow the assessment of large quantities of phenotypic data under controlled environments [4,5,6,7,8,9]. Although these systems enable a detailed non-invasive plant assessment throughout the plant life cycle under controlled conditions, they neglect information about the genotype-environment interactions and do not take horticultural or viticultural plants into account. However, grapevine, for example, as a rather large perennial plant, needs to be evaluated directly in the field. Several studies of the implementation of new techniques for an improved management of vineyards in practical viticulture [10,11,12,13,14] have been conducted in recent years. Yield estimation is one of the most important traits in precision viticulture due to annual and spatial variations. The published studies aimed to improve yield estimation and forecasting by detecting bunches of grapes, berries [15,16,17,18] or the number of inflorescences [19] in images. Ground-based sensor data used in precision viticulture are than either recoded from a constant distance to the canopy [16,19,20,21], mounted to a tractor [10,11,12], truck crane [22] or include modified vehicles [13,15,23] equipped with global positioning systems (GPS) devices [18,24,25]. Another approach is the application of a field phenotyping robot. Such systems have already been introduced for application in maize [26] and small grain cereals [27]. A robot application for viticulture was suggested by Longo *et al.* [28]. The U-Go (Unmanned Ground Outdoor) robot was developed as a multipurpose vehicle with the aim of facilitating work during the season (harvesting, pruning, transportation of bins) [28]. Furthermore, the opportunity to be equipped with a modular remote sprayer [29] is given. Its technical specification allows remote control or autonomous motion using GPS waypoints [28]. Nonetheless, all of these studies focus mainly on vineyard management, site-specific information to improve crop load, water or the health status of the considered plot. In contrast, grapevine breeding aims at the phenotyping of single grapevines, whereby genetic resources and large sets of breeding material need to be screened. That implies that in one experimental field plot, each plant can be a different genotype, showing its distinct phenotype, which needs to be assessed individually with high precision. Not only the resolution of phenotypic data towards one single grapevine may differ, also the variation of traits within breeding material is considerably higher than in commercial vineyards. Important phenotypic traits in grapevine breeding are the detection of fruit parameters, e.g., the berry size and color of berries. Current assessment of phenotypes in breeding programs relies largely on visual estimations, using the BBCH (phenological development stages of a plant; stands for Biologische Bundesanstalt, Bundessortenamt und CHemische Industrie) scale [30] or OIV (International Organization of Vine and Wine) descriptors [31]. These systems are laborious, time-consuming and, therefore, expensive. The data obtained are subjective and can vary significantly when evaluated by different persons. The biggest limitation, however, is the needed simultaneous screening of vines from several hectares of experimental vineyards, which limits a detailed evaluation of traits to a rather small number of breeding strains. The application of non-invasive, high-throughput sensor technologies is required to increase the efficiency of grapevine breeding by increasing the phenotyping efficiency (number of plants per time), improving the quality of phenotypic data recording and reducing the error variation. Such new methods progressively increase the amount of data that needs to be handled.

First steps towards a high-throughput phenotyping pipeline in grapevine breeding have been introduced by Herzog *et al.* [32]. The study implemented a Prototype Image Acquisition System (PIAS) for semi-automated capturing of geo-referenced images and a semi-automated image analysis tool to phenotype berry size. An automated phenotyping platform in grapevine breeding is needed to screen for phenotypic traits on a single-plant-level in a reasonable time, unlike the application in precision farming, whereas the overall appearance of a plot or at least single areas of a plot are of greatest interest.

Here, we describe the setup of an updated and expanded phenotyping pipeline involving automated data acquisition in the field, automated data management and data analysis. The challenges of this pipeline are the combination of: (1) automated simultaneous triggering of all cameras at a predefined position in the field; (2) automated acquisition of geo-referenced images; (3) data management via a database; and (4) automated image analysis for objective and precise phenotyping of the berry size and color. Moreover, we demonstrate the application of the pipeline in the grapevine repository at Geilweilerhof.

## 2. Material and Methods 

### 2.1. Plant Material

The application of the phenotyping pipeline involved 2700 grapevines representing 970 accessions from the grapevine repository at the experimental vineyards of Geilweilerhof located in Siebeldingen, Germany (N 49°21.747, E 8°04.678). Interrow distance was 2.0 m, and grapevine spacing was 1.0 m. Rows were planted in a north-south direction. Colored size reference labels were fixed to the wires and used to scale the images.

### 2.2. Automated Image Acquisition

**Figure 1:**
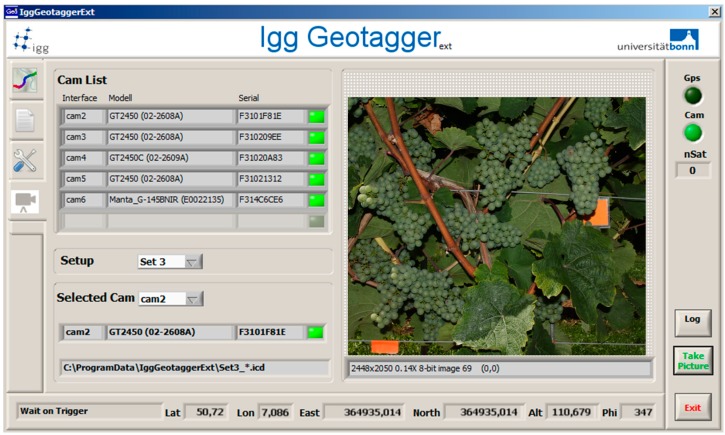
Graphical user interface of the *IggGeotagger.Ext*. The software manages the communication between the control unit of the *PHENObot* and the image acquisition PC, triggers the cameras and controls the image transport and storage. It is preferentially used for the visualization of captured images and for setting the camera parameters.

**Figure 2:**
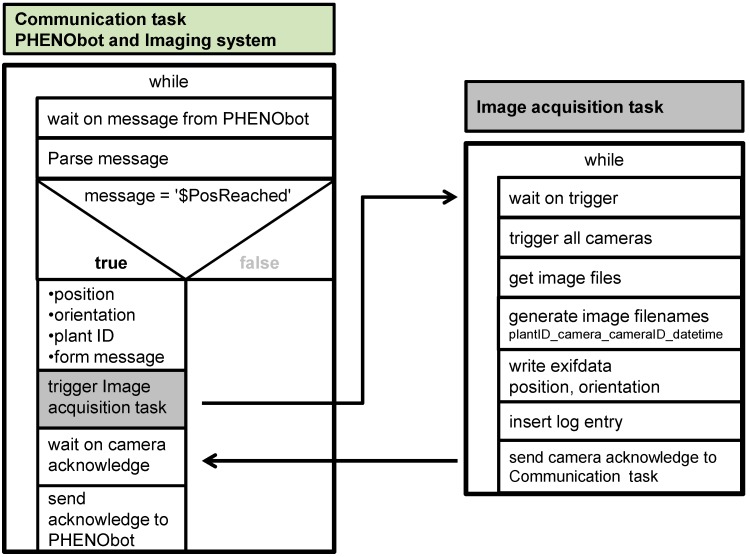
Communication and image acquisition task within the *IggGeotagger.Ext* software. The communication task handles the communication between the control unit of the *PHENObot* and the image acquisition PC; the image acquisition task controls the cameras and the image transport and storage.

For the automated image acquisition directly in the field, the *PHENObot* (Phenotyping robot) was developed [33]. This phenotyping platform consists of a chain vehicle containing a control unit and a camera-light unit in combination with an industrial computer. In order to operate in a harsh outdoor environment and to enable the transportation and navigation of the camera-light unit for the non-destructive inspection of phenotypic grapevine traits, the chain vehicle had to meet certain requirements: a lifting capability up to 250 kg, low vibration drive at a speed between 4 to 6 km·h^−1^, an easily adjustable mounting system for the sensors, a navigation system based on GPS coordinates, the ability for path planning, as well as fulfilling safety standards [33]. For targeted image acquisition, path planning is needed for the *PHENObot*. Therefore, precise GPS positions of individual vines are necessary and, so, all grapevines have been surveyed. The camera-light unit used on the *PHENObot* consists of three monochrome cameras (AVT GT-2450; objective: CVO 8 mm; 2448 × 2050 pixels), one RGB camera (AVT GT-2450C; objective: Schneider KMP-IR CINEGON 8 mm; 2448 × 2050 pixels) and one NIR camera (AVT MANTA; objective: Schneider KMP-IR CINEGON 8 mm; 1388 × 1038). To enable an adequate illumination for standardized image acquisition, a lightning unit containing eight LED bars (12 LEDs; ODLW300 series; Smart vision lights, Muskegon, MI, USA) was combined with the camera unit (for the setup, see Figure 1A). The components are connected with the image acquisition computer by a fast Ethernet network (GigE). All cameras are synchronously triggered using this network, and the images are transmitted immediately to the PC. The lightning unit is triggered by one of the monochrome cameras. For configuration and monitoring of the image acquisition process, a software application (*IggGeotagger.Ext*) has been developed fulfilling two main tasks: the communication task handles the communication between the control unit of the *PHENObot* and the image acquisition computer; the image acquisition task controls the cameras and the image transport and storage. The application is also used for visualization of the images and for setting the camera parameters (screenshot in Figure 1). A single image acquisition cycle performs several steps (see Figure 2). The communication task waits for a message from the *PHENObot* control unit. As soon as the *PHENObot* has reached a predefined position, it sends a specific message containing the position, the orientation and the corresponding plant ID to the computer. Then the communication task starts the image acquisition task, which triggers all cameras, receives the images, generates the filenames for the images (plantID_camera_cameraID_datetime) and saves them to the hard drive. Additionally, the position and orientation information is written directly into the file header of the image. When the image acquisition task has finished, the communication task sends an acknowledgment message to the *PHENObot* control, signaling that it can move to the next position. One hundred forty grapevines have been assessed to verify the image section: (1) includes the whole bunch area of each grapevine assessed, and (2) remains the same when repeatedly approached. The *PHENObot* was stopped at the surveyed position of the grapevine and under the consideration of the training direction (trained to the south or north, respectively). Moreover, the 140 grapevines have been approached 4 times in a row.

### 2.3. Data Management

All 2700 grapevines of the genetic repository have been surveyed using a RTK (real-time-kinematic)-GPS system (Trimble^®^ SPS852, Geo Systems GmbH, Jena, Germany) with 2-cm accuracy. The geo-information of each grapevine and the associated plant ID is stored in the central database, *PLA* (*Plant Location Administration*)—A common management tool for experimental areas in the Julius Kühn-Institut. All images delivered by the *IggGeotagger.Ext* are imported into the database, *IMAGEdata*. Based on the image names, which contain the plant ID, every image is uniquely assigned to a single grapevine. For this assignment, the *PLA* is used. *PLA*, as well as *IMAGEdata* work with geographical data (UTM). The aim of *IMAGEdata* is to have a powerful and easy to use tool for managing the images as a basis for further evaluation. These databases can be used by modern Web 2.0 interfaces and web services. Current technologies allow safe operation and offer modern user interfaces.

### 2.4. Image Analysis

Image analysis was conducted by using the MATLAB^®^-based tool, *BIVcolor* (Berries in Vineyards-color). Based on a one-class classification framework determining grapevine berry sizes, some slight modifications have been done (MATLAB 2012b and Image Processing Toolbox, The Mathworks, Natick, MA, USA) on the Berries in Vineyards (*BIV*) algorithm [34]. This was targeted to separately record mean RGB values of each single berry according to their color channels (RGB) and their position within the corresponding image. The data were written loop-wise into a tab-limited text file corresponding to the image file analyzed and finally stored in a SQL-database (Access 2010, Microsoft, Redmond, WA, USA). The known position of berries within a trait later on provides clustering to check berry patterns and outliers.

A set of 500 images, including 235 different accessions and *n* = 1,300,900 segmented single berries, was used for color information assessment. The mean of the RGB values of all berries detected in one image were used for statistical analysis. As reference data, the berry color was assessed as five classes (1 = black; 2 = red; 3 = rose; 4 = grey; 5 = green).

### 2.5. Statistical Analysis

Statistical analysis was conducted using the software R Version 3.1.1 (R Foundation for Statistical Computing, Vienna, Austria). Linear discriminate analysis (LDA) was performed to predict the berry color class using the RGB values as predictor variables.

## 3. Results and Discussion

### 3.1. Field Application of the Phenotyping Robot

**Figure 3:**
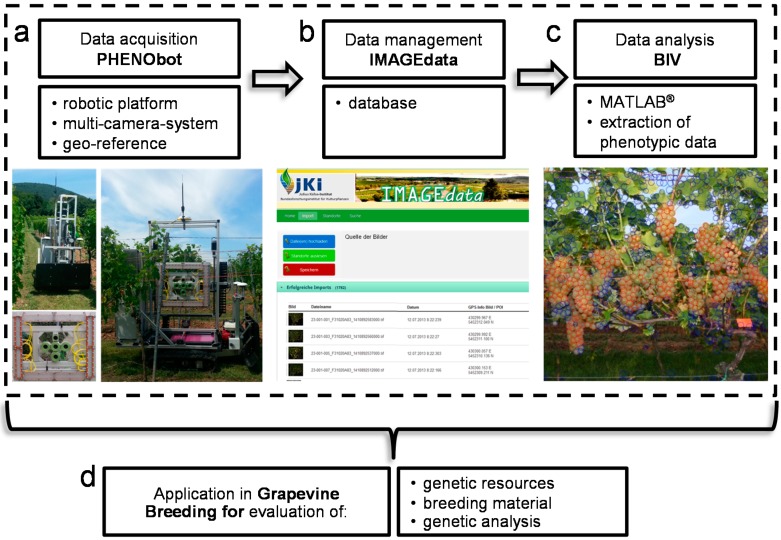
Phenotyping pipeline in grapevine breeding. (**a**) Data acquisition using the *PHENObot* consisting of a robotic platform, a multi-camera-system and a geo-information system; (**b**) data management of the sensor data is achieved by a database (*IMAGEdata*); (**c**) data analysis through the application of MATLAB^®^-based tools, e.g., *BIVcolor* (Berry in Vineyards-color), to extract the phenotypic data; (**d**) the phenotyping pipeline was developed for application in grapevine breeding. This enables the phenotyping of large sets of plant material from genetic resources or breeding material.

**Figure 4:**
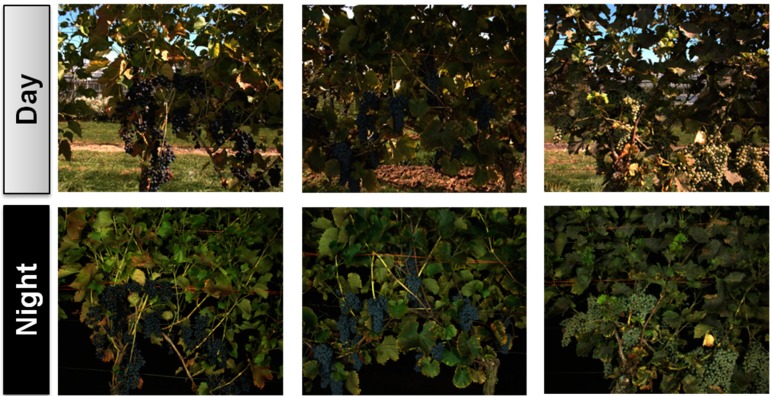
Comparison of images taken during the day and at night. Three examples of vines photographed on a sunny day and at night. All images were captured using the *PHENObot* with the lightning unit on. Image acquisition at night enables standardized conditions, which are very important for robust automated image analysis and comparable phenotyping results, e.g., with regard to the determination of berry colors.

A phenotyping pipeline has been set up and consists of the following components: (1) data acquisition; (2) data management; and (3) data analysis (Figure 3). Data acquisition was done automatically using the *PHENObot*. Each image was linked to one plant, respectively one genotype, without any post-processing. Applying the *PHENObot* image data from 2700 grapevines representing 970 grapevine accessions has been done. Automated data recording for these large set of plants was completed within 12 h. The image acquisition of one grapevine took on average 15 s. Although the camera was equipped with a lightning unit, it was impossible to take standardized images on sunny days (Figure 4). Consequently, the image acquisition in the grapevine repository was done at night due to uniform light conditions. This has also been reported to work best for images taken in commercial vineyards to estimate yield [18].

Two pre-test drives consisting of 140 grapevines have been done. The first one to ascertain the image section comprises the whole bunch zone of each grapevine assessed and the second one to make sure the same image section is captured each time a grapevine is approached. The image section was best when the stopping position of the *PHENObot* was shifted 25 cm south or north in accordance with the training direction in order to enable one to see as much of the bunch zone as possible. The 140 grapevines were approached four times, and the image section stayed the same for each grapevine and all four repetitions. The comparison of the GPS position logged at the image acquisition point for the four drives showed a difference of 1–2 cm, which is within the accuracy of the GPS system.

### 3.2. Image Analysis

**Figure 5:**
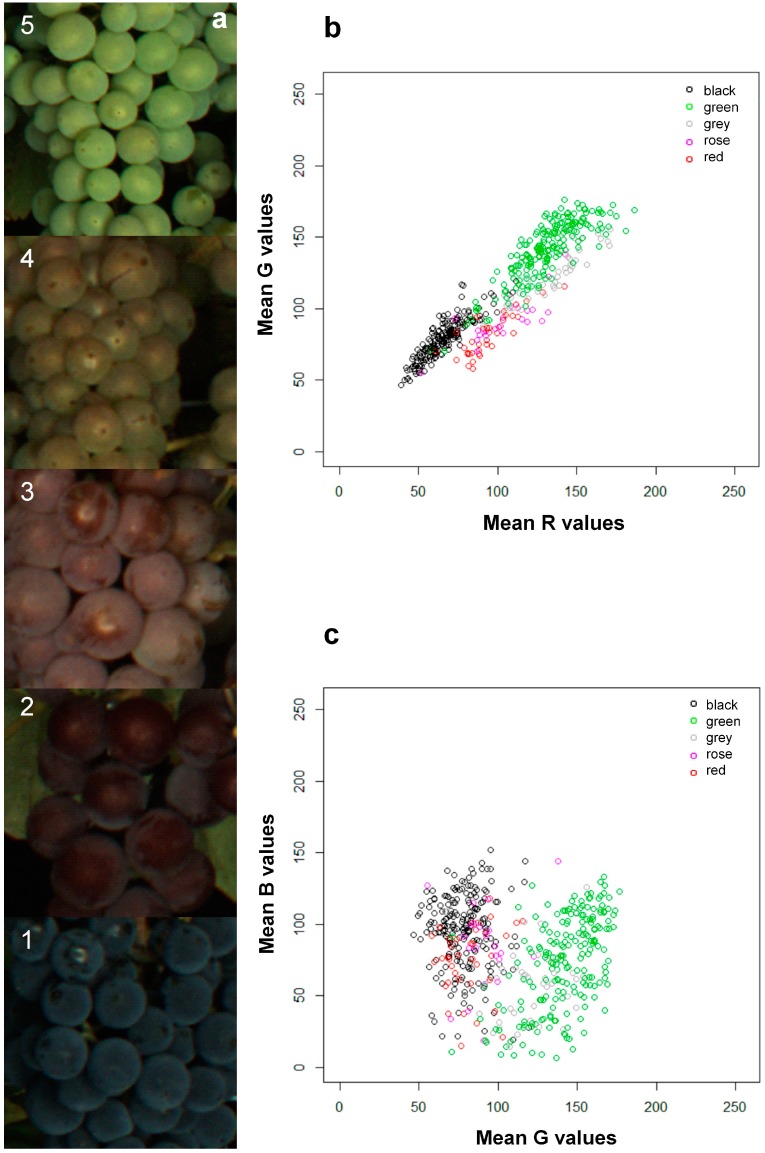
Distance plots of single RGB values indicating the fitness of the color model used for LDA. Prediction of berry color classes was done using the image-based detected RGB values. LDA used three parameters (red, green and blue color values) and, as the ground truth, the visually assessed berry color. (**a**) Berry color was visually assessed as five classes: Class 1 = black; Class 2 = red; Class 3 = rose; Class 4 = grey; Class 5 = green; (**b**) distance plot of R values *vs.* G values; (**c**) distance plot of G values *vs.* B values.

Images have been analyzed using the MATLAB^®^-based tool, *BIVcolor*. The tool enables the automated extraction of the phenotypic traits, berry size and color. The berry size is one of the most important fruit parameters integrated for seedling selection in breeding programs. The *BIVcolor* evaluated berry size ranging from 9.8 mm to 13.9 mm. The acquisition of the berry color is important for the characterization of genetic repositories or the phenotyping of mapping populations for genetic analysis. Initially, the color of grapes can be classified according to the presence or absence of anthocyanin in the berry skin, as either black or green. As a result of natural hybridization and human selection, the grape skin color is very diverse nowadays, ranging from green-yellow, grey, rose, red to black. The reference assessment for berry color in the set of 500 images showed a distribution of: 202 (Class 1 = black), 200 (Class 5 = green), 39 (Class 4 = grey), 37 (Class 2 = red) and 22 (Class 3 = rose) (Figure 5a). Linear discriminant analysis (LDA) using three predictor variables (red, green and blue color values) was used to predict the class of berry color. Table 1 shows the cross-validation of the real *vs.* predicted color class. The percentage of the correct prediction of black (197 berries; 97%) and green (178 berries; 89%) berries was very high. Some of the green berries were predicted as grey, but in most cases, grey berries were predicted as grey (28 berries; 71%). Thirteen images (59%) visually assessed as rose berries have been predicted as red. The difference between red and rose berries can be difficult to discern no matter whether one predicts the class doing visual estimations (Figure 5a) or if one uses RGB values (Figure 5b,c). Due to the fact that RGB values of these two classes are very similar and overlapping (Figure 5b,c), it was not possible to distinguish these two classes in our study. One can clearly distinguish between black, green, grey and red/rose berries, and this is exactly what can be used for the evaluation of genetic resources and breeding material, but also for the management of grapevine repositories. Usually, three grapevines of one accession are planted next to each other, through the image-based color detection planting mistakes based on wrong berry color can be uncovered, for instance.

**Table 1:** Cross-validation of the real berry color classes assessed by visual estimation and the color classes predicted with the LDA.

	Real Color Classes				
Predicted Color Classes	Black	Green	Grey	Red	Rose
black	197	7	2	5	3
green	5	178	7	0	0
grey	0	15	28	2	3
red	0	0	1	26	13
rose	0	0	1	4	3

From previous work presented by Roscher *et al.* [34], it is known that the acquisition of images in the field and automated image analysis in order to determine berry sizes is about 24-times faster compared to the application of a caliper to measure the diameter of 50 berries per grapevine. The image analysis runs automatically and needs no user interaction after starting the program. Thus, the analysis can be performed simultaneously as daily work within the common breeding program. With the extension of the *BIV* tool [34] to *BIVcolor*, we gained information about an additional phenotypic trait that can be extracted from the images without losing any time for evaluations. Another advantage is that images can always be analyzed retrospectively when new tools come along.

### 3.3. Future Work

The phenotyping pipeline has been successfully tested in grapevine breeding. So far, only the RGB images are used for automated image analysis. The camera unit consisting of five cameras (one RGB, three monochrome and one NIR camera) offers more opportunities. It enables the generation of 3D information using the monochrome cameras [32]; furthermore, it is suitable to use the NIR information for vitality indices. In addition, it is conceivable that the sensor unit of the PHENObot is going to be extended by additional sensors, like lasers, multi- or hyper-spectral sensors. There are plans to connect the *IMAGEdata* database with other existing databases, like VIVC (Vitis International Variety Catalogue [35]) and the European Vitis Database [36], to complete the linkage of available information.

An important stage in grapevine development is the beginning of berry ripening, namely veraison. This is the time when the berries start to soften and colored cultivars start to change their color, e.g., from green to black. It is conceivable that *BIVcolor* can be used to detect that date if images are taken continuously throughout the growing period.

## 4. Conclusions

A setup of a phenotyping pipeline has been introduced for grapevine breeding and to support the management of a grapevine repository. A robotic platform, the *PHENObot*, was built to enable the automatic image acquisition directly in the field. In order to facilitate the management of the data gained by automated image acquisition, an image database was developed. Compared to human visual assessments, a larger set of grapevines can be screened automatically, and the data revealed are objective and precise.

## Appendix

To be able to reproduce results and conclusions, a set of 100 RGB images and the associated results from *BIVcolor* were deposited and are freely accessible at the data repository of the Julius Kühn-Institut, the Federal Research Centre for Cultivated Plant in Germany (doi:10.5073/jki-data.2015.1).
